# Pride-Based Self-Affirmations and Parenting Programs

**DOI:** 10.3389/fpsyg.2020.00910

**Published:** 2020-06-23

**Authors:** Zoelene Hill, Michelle Spiegel, Lisa A. Gennetian

**Affiliations:** ^1^The Institute of Human Development and Social Change, New York University, New York, NY, United States; ^2^School of Education, University of California, Irvine, Irvine, CA, United States; ^3^Sanford School of Public Policy, Duke University, Durham, NC, United States

**Keywords:** self-affirmation, parent engagement, prevention, parenting intervention, behavioral economics, racial disparity, ethnic disparity, socioeconomic disparity

## Abstract

We newly apply the concept of self-affirmation typically used in the domain of health and education to the domain of parenting. Recruiting parents of children age 13 or younger (*n* = 1,044), we test how eliciting positive self-concept affects interest in receiving parenting materials and participating in a parenting program. We find that an adapted, pride-based written self-affirmation exercise increased parents’ positive self-concept and their interest in parenting programs and resources, particularly among parents with a high baseline fear of judgment associated with seeking help. Implications for applying insights from psychology as a strategy to mitigate fear of judgment to optimize participation in an evidence-based parenting program at scale are discussed.

## Introduction

Self-affirmations are self-generated thoughts or experiences that bring about an expanded and positive view of the self and can reduce resistance to situations perceived as threatening (Steele, 1988; McQueen and Klein, 2006; Sherman and Cohen, 2006). Self-affirmation exercises typically solicit reflection from the respondent through various writing exercises (McQueen and Klein, 2006). They have been shown effective across contexts including academic performance of historically marginalized student groups (Cohen et al., 2006), achievement of women in STEM courses (Miyake et al., 2010), and adolescents’ prosocial behavior (Thomaes et al., 2012). Self-affirmations have been found to increase individuals’ openness to receiving threatening information about their health (Harris and Epton, 2009) and to increase health-promotive behaviors such as adhering to prescription dosage (Ogedegbe et al., 2012; Cohen and Sherman, 2014). While the influence of self-affirmation has been demonstrated in various contexts, it has not been examined in the context of parenting support.

Providing parenting education, information, and programming support is an increasingly popular model for promoting child development. For example, parenting support is a long-standing feature of the federal Head Start program, which serves over 1 million children and families annually (Administration for Children and Families, 2019). However, the value of this approach hinges on equipping early care and education systems with tools to optimize parent participation. Fear of judgment can reduce parents’ openness to receiving information and education about their parenting and can decrease their willingness to participate (Koertig et al., 2013; Mytton et al., 2014), thereby reducing the intended family-level and community-level impacts of public investments in early education initiatives.

To test whether written self-affirmation exercises have the potential to support parents’ interest in such programs, we adapt and experimentally test a pride-based self-affirmation among over 1,000 parents recruited online (Klein et al., 2001; Hall et al., 2014). Disarming fear of judgment for one’s parenting through self-affirmation is hypothesized to increase parents’ interest in receiving parenting materials and participating in a parenting support program. The current study addresses these research questions: How does a pride-based self-affirmation exercise affect parent interest in parenting programs and resources? How does it affect hypothesized mediating outcomes? How does it differ for mothers versus fathers, for parents of younger children (birth to 5 years) versus older children (6 to 13 years), for families with lower versus higher income, by race and ethnicity, and by existing fear of judgment about seeking help with parenting?

## Fear of Judgment as a Barrier to Seeking Parenting Support

Fear of judgment can affect choices during decision-making junctures, posing a barrier to interest and uptake of parenting support even among parents who want it. Indeed, meta-analyses suggest that parents fear judgment from others for seeking parenting support and feel reluctant to signal deficiency by revealing parenting challenges (Koertig et al., 2013; Mytton et al., 2014). Empirical evidence indicates that fear of judgment is a salient barrier to actual participation in parenting programs (Taylor et al., 2013), whereas lower fear of judgment is associated with greater likelihood of participating (Dempster et al., 2013). In addition, fear of judgment for seeking support is a rational response to daily lived experiences of racism and discrimination and may help explain why low-income parents and parents of color avoid parenting programs (Schultz and Vaughn, 1999; Keller and McDade, 2000; Sirey et al., 2014). Parents also convey how the stresses of poverty can undermine positive self-concept: “I got caught up in thinking that if I can’t bring the income in, then I am not really a great parent” (Russell et al., 2008, p. 88).

One way to alter the context at decision-making junctures to help curtail avoidant responses and harness an approach orientation to threats (versus avoidance, in the parlance of Cohen and Sherman, 2014) is self-affirmation. The role of self-affirmations in optimizing participation in parenting programs has not yet been examined. However, in a highly relevant application, a positive affirmation designed for overcoming negative self-concept associated with poverty (specifically, food deprivation and security) increased individuals’ expressed interest in social assistance relative to those randomly assigned to a neutral condition (Hall et al., 2014).

In our proposed theory of change, psychological barriers, including the fear of judgment and reluctance to signal difficulties with parenting associated with participating in a parenting program, may threaten parents’ sense of self and interfere with program uptake and participation. The contexts of poverty and racism can heighten feelings of being threatened or targeted as incompetent. Self-affirmations remind parents of positive self-concept and values beyond the threatened domain—in this case parenting—and support a constructive, approach-oriented response to the invitation to receive parenting support.

## Current Study

We tested the effect of a pride-based self-affirmation exercise on parents’ interest in learning more about and attending parenting programs and parents’ decision to receive parenting information and support. We tested the effect on two hypothesized mediating outcomes: self-concept and fear of judgment for help-seeking. We examined how the effect of the self-affirmation differs by select participant subgroups. We utilized an online platform to efficiently recruit a sample of parents to rapidly test the application of self-affirmation to decision-making about parenting support.

Participants were asked to think about a time when they felt successful or proud and to respond to three reflective questions about the chosen experience. Specifically, these questions were “What did you do that you were proud of?,” “What motivated you?,” and “What led to your success?” The pride-based written exercise was adapted from Hall et al. (2014). Respondents who received the neutral writing prompt were asked to describe their most recent meal and to answer three reflective questions about the meal. These reflective questions were “Describe what you had to eat,” “How did you choose what meal you would eat?,” and “How was your meal prepared?” Our outcome variables—parent interest in learning more about and attending parenting programs and parent decision to receive parenting information and support—are conceptualized as a stepping stone to subsequent participation (Ajzen, 1991). Consistent with previous studies of self-affirmation, we hypothesize that parents receiving the pride-based self-affirmation exercise will express a higher level of interest in parenting programs and resources. Further, acknowledging the complexity of behavior (MacKinnon, 2011; Kenny, 2018), we examine how select participant characteristics moderate the causal relationship between self-affirmation and our outcomes, with the effect hypothesized to be strengthened among parents of young children (who have less parenting experience); racial and ethnic minority parents, low-income parents, and parents who have trouble meeting monthly expenses (all of whom experience greater social stigma); mothers (who bear a larger share of the caregiving burden); and those with higher levels of existing fear of judgment for help-seeking.

## Method

We tested an adapted form of a pride-based self-affirmation exercise (Hall et al., 2014). To avoid possible imbalanced attrition due to the time burden of writing, both the treatment and control groups were prompted to write short responses to the same number of items. Approximately half (*n* = 506) of the respondents who consented and initiated responses to an online survey randomly received a pride-based self-affirmation exercise, while the others received a neutral writing prompt (control group). We analyze main effects on targeted outcomes and the hypothesized mediating outcomes and conduct moderation analyses using select sample characteristics.

### Sample and Data

We recruited parents online using PrimePanels, an advanced version of Amazon Mechanical Turk intended for scientific research, in which eligible participants are prescreened based on demographic characteristics. Aspiring participants are first asked demographic questions at random times, not connected to any specific survey, so there is minimal (if any) incentive to falsify characteristics. PrimePanels follows up with aspiring participants weeks or months later, asking them the same or similar demographic questions for updated information. PrimePanels reconciles the two questionnaires, and once consistency is determined, participants are approved. PrimePanels then displays relevant surveys (anonymously) to targeted participants based on researchers’ needs.

Prescreened participants were shown a link on their PrimePanels dashboard to our Qualtrics survey. In the survey, participants were first asked for their consent to participate. Those who consented completed the survey. Respondents were paid a portion of $3.15, the PrimePanels price per respondent, for completing the survey.

We recruited a sample of 1,228 parents of children age 13 or younger. Using a PrimePanels filter, we limited the United States regions from which our sample was drawn to the south and northeast to flexibly allow follow-up and extension studies using PrimePanels with reduced threat of resampling the same parents. To help ensure validity of information about the parent status of participants recruited online, we embedded a screener within the survey in which respondents were asked the ages of their children. The final analytic sample for the full study consisted of 1,044 participants. Of the 184 excluded from the full study, 19 did not consent to participate; the remaining 165 were excluded because of missing data for a covariate or outcome variable but did not qualitatively differ from the responding sample.

### Measures

All participants were asked a series of questions regarding their children and their household and individual demographic and socioeconomic characteristics and rated their level of interest in parenting information and programming.

#### Parent Interest in Learning More About and Attending Parenting Programs

Parents were asked to rate their response on a scale of 0 (“not interested at all”) to 100 (“very interested”) to the questions “If there was a program in your local community about how to be a better parent, how interested would you be in learning more about this program?” and “How interested would you be in attending this program?” The overall mean score across conditions for interest in learning more about the program was 63.04 (*SD* = 27.35). The mean score for interest in attending the program was 57.82 (*SD* = 28.15).

#### Parent Decision to Receive Parenting Information and Support

Parents were asked, “Would you like to receive information about parenting support online resources? If yes, please provide your email or cell phone number below,” as a measure of an actual behavior.

#### Self-Concept

Parents were asked to rate how they feel about themselves on a scale of 0 (not positive) to 100 (extremely positive), with 50 labeled as “neutral,” on the item “How do you feel about yourself right now?” immediately after the experimental task (affirmation or neutral writing task). This scale was initially used by Hall et al. (2014).

#### Fear of Judgment for Help-Seeking

We used a previously validated 10-item self-report scale adapted from Vogel et al. (2006), which seeks to measure the construct of fear of judgment or “self-stigma” for psychological help-seeking (e.g., “I would feel inadequate if I went to a therapist for psychological help”). In our adaptation, we replaced “therapist” and “psychological help” with the relevant context (e.g., “I would feel inadequate if I went to a parent support program for help”). We administered this scale to a random half of the total sample (*n* = 512) primarily to preserve the other random half of the subsample (*n* = 532) from possible confounding influences. Furthermore, within the subsample who received the scale, we randomized half to receive the scale before the experimental task (*n* = 263) and the other half to receive the scale after the task (*n* = 256). Following the original scale scoring procedure, we scored each item and summed the item scores, with higher total scores indicating higher levels of fear of judgment for help-seeking (*M* = 22.86; *SD* = 6.371). The score is symmetrically distributed, with a median score of 22. For ease of interpretation in the moderation analysis, we divide the sample to create groups with above-average (*M* = 28.13; *SD* = 4.90; *n* = 235) and below-average (*M* = 18.38; *SD* = 3.32; *n* = 277) fear of judgment for help-seeking.

#### Household Income and Meeting Monthly Living Expenses

Participants were asked, “What is your estimated annual household income including all who contribute to income in the household?” Categorical response options started with “less than $5,000” and increased by $5,000 increments up to “$150,000 and over.” As sample sizes in each of the 16 income categories were insufficient and these categories had no substantive meaning, we recategorized the income variable into “low” (below $29,000), “middle” ($30,000–$74,999), and “high” ($75,000 and above) incomes. These categories map onto national distributions of income (the low category is at or lower than 100 to 150% of the federal poverty level, the middle category reflects roughly the median income in the United States, and the high category reflects higher income). Additionally, the terciles empirically represent the data’s distribution.

Subsequently, participants were asked, “During a typical month, how often do you worry about being able to meet monthly living expenses?” Categorical response options ranged from “never” to “always.” We combined the “frequently” and “always” respondents to create three categories for a split sample analysis (“always or frequently has trouble,” “sometimes has trouble,” “never has trouble”). Among low-income parents, 46.6% reported always or frequently having trouble meeting monthly expenses compared with 30.8% of parents in the middle-income category and 17.6% of parents in the high-income category. Generally, respondents across all income categories reported sometimes having trouble meeting monthly expenses (53% in the middle-income category and 44.9% in the high-income category).

#### Demographic Characteristics

Table 1 presents demographic characteristics of the full study sample. The majority of parents were married at the time of the survey (71.7%), comparable to national estimates of children under 18 residing with married parents. Most respondents reported working full-time (60.8%). In terms of education, 28.1% of fathers had completed high school or less, and 19.1% of mothers had completed high school or less. Approximately 11% of the sample identified as black, slightly less than the national population of 13 and 9% of the sample identified as Hispanic, less than the national population of 18% (United States Census Bureau, 2018).

**TABLE 1:** Selected descriptive characteristics of the sample.

Characteristics	Percentage
**Relationship status**	
Currently married	71.7%
Divorced	6.1%
Living with a partner	10.7%
Never married	7.8%
Single, no partner	2.7%
Widowed	1.0%
**Work status**	
Not working, looking for work	7.7%
Not working, not looking for work	19.8%
Work full-time	60.8%
Work part-time	11.8%
**Mother’s educational attainment^a^**	
High school or less	19.1%
Any college up to 4-year degree	62.3%
More than 4-year college	18.6%
**Father’s educational attainment^b^**	
High school or less	28.1%
Any college up to 4-year degree	55.4%
More than 4-year college	16.5%
**Race/ethnicity**	
Hispanic	9.4%
Black	10.8%
White	73.8%
Other	6.0%
**Region^c^**	
West	0.2%
South	50.9%
Midwest	0.3%
Northeast	48.6%
Puerto Rico	0.1%
**Number of children**	
1	34.5%
2	41.5%
3	14.8%
4	6.0%
5 or more	3.3%

*Authors’ calculations from data collected via participant responses. *N* = 1,044. ^*a*^*n* = 634 mothers. ^*b*^*n* = 375 fathers. ^*c*^As designed, the sample represents 25 states in the northeast and south.*

### Analytic Approach

For the main effects, intent-to-treat estimates of the randomized self-affirmation treatment were examined *via* simple regression analyses of the form *y_i_* = β*Exp_i_* + ε*_i_*, where for each parent respondent *i*, *y* represents the main outcomes and hypothesized mediators described earlier, and β represents the impact of the pride-based self-affirmation treatment. The intent-to-treat estimates were obtained separately within each level of the moderator, allowing for an unrestricted moderation analysis (Weiss et al., 2013).

## Results

### Main Effect of the Impact of the Pride-Based Self-Affirmation

Table 2 shows that, as hypothesized, the pride-based self-affirmation increased the level of interest in both learning more about parenting programs and attending parenting programs by approximately 6 percentage points (*p* = 0.001). Parents in the control group rated their interest in learning more about parenting programs at 60.26, while parents in the treatment group rated their interest at 66.0 (*p* = 0.001). Parents in the control group rated their interest in attending parenting programs at 54.98, while parents in the treatment group rated their interest at 60.86 (*p* = 0.001). Lastly, we analyzed the effect of the self-affirmation on behavior. Although not statistically significant (*p* = 0.17), parents in the treatment group were more likely to submit their email address to receive information about online parenting support resources than parents in the control group, with 42% of the control group and 46% of the treatment group submitting their email address.

**TABLE 2:** Main effects of a pride-based affirmation on interest in parenting programs.

		Interest in learning more about a parenting program			Interest in attending a parenting program			Provided email to receive information about parenting support online resources		
	*n*	Unadjusted control group mean	β	*p*-value	Unadjusted control group mean	β	*p*-value	Unadjusted control group	β	*p*-value
Full sample	1,044	60.26	5.74**	0.001	54.98	5.88**	0.001	0.42	0.04	0.17
			(1.69)			(1.73)			(0.03)	

Authors’ calculations from data collected via participant responses to PrimePanels in April 2018. Beta (β) represents the coefficient from an ordinary least squares regression estimating the impact of receiving the pride-based affirmation on the outcome. Standard errors of the coeffients are in parentheses; unadjusted control group means are based on items with scaled (0–100) responses. **p < 0.01.

### Effects of the Pride-Based Self-Affirmation on Hypothesized Mediators

Table 3 shows results of the manipulation checks. We randomized the participants into subsamples who received the fear of judgment scale either before or after the self-affirmation exercise or neutral task. A two-way ANOVA test indicates that there is no statistically significant relationship between the fear of judgment scale score, the experimental group, or timing (i.e., before or after writing prompt) of receipt of the fear of judgment scale. Table 3 reports on the subsample who received the scale after the self-affirmation or neutral task (*n* = 256), showing no statistical differences in the mean fear of judgment scale score by treatment status. We regress the two hypothesized mediators, self-concept and fear of judgment, on the three outcomes of interest and find suggestive evidence that self-concept acts as a mediator. Respondents who received the affirmation experience a boost in self-concept (β = 4.24, *p* < 0.001). Further, positive self-concept predicts higher interest in learning more about (β = 0.29, *p* < 0.001) and attending programs (β = 0.28, *p* < 0.001); and the percent of parents submitting their email addresses slightly increases (β = 0.002; *p* = 0.055). While we find that fear of judgment predicts decreased interest in learning more about (β = −1.19, *p* < 0.001) and attending programs (β = −1.28, *p* < 0.001), and the percent of parents submitting their email address decreases (β = −0.017, *p* < 0.001), we were unable to detect a relationship between the affirmation and fear of judgment scores, which suggests that, while self-concept is a mediator, fear of judgment is not.

**TABLE 3:** Hypothesized mediators of the impact of a pride-based affirmation on interest in parenting programs.

		How do you feel about yourself right now?				Fear of judgment for seeking parenting help^Δ^		
	*n*	Unadjusted control group mean	β	*p*-value	*n*	Unadjusted control group mean	β	*p*-value
**Full sample**	1,044	71.91	4.25*** (1.15)	0.01	256	22.66	−0.22 (0.80)	0.79
**By household income^a^**								
Low	161	66.40	2.94 (3.45)	0.40				
Medium	400	72.40	5.76** (1.84)	0.002				
High	483	73.01	4.21** (1.57)	0.01				
**By relationship to child**								
Mother	634	68.43	6.02*** (1.51)	0.001				
Father	375	76.04	3.12 (1.77)	0.08				
**By child age**								
Has child below age 5	498	70.67	4.71** (1.67)	0.01				
Does not have child below age 5	532	72.95	3.87* (1.61)	0.02				
**By fear of judgment for help-seeking^b^**								
Low	277	77.78	−0.84 (1.96)	0.67				
High	235	68.44	7.91** (2.30)	0.010				
**By trouble meeting monthly expenses^c^**								
Always or frequently has trouble	283	64.55	5.22* (2.59)	0.05				
Sometimes has trouble	501	72.18	5.38*** (1.51)	0.00				
Never has trouble	260	78.14	3.33 (1.92)	0.14				
**By race/ethnicity**								
White	770	70.02	5.08*** (1.32)	0.00				
Black, Hispanic/Latino, Other	274	77.26	1.90 (2.27)	0.41				

Standard errors of the coefficients are in parentheses; unadjusted control group means are based on items with scaled (0–100) responses. *p < 0.05; **p < 0.01; and ***p < 0.001. Beta (β) represents the coefficient from an OLS regression estimating the impact of receiving the pride-based affirmation on the outcome. ^Δ^Calculated using the 25% of the sample randomized to receive the fear of judgment scale after the randomized affirmation or control task. ^*a*^Household income does not account for household size. Low income < $30,000; medium income $30,000; and ≤$74,999 high income. ^*b*^Fear of judgment is measured via a 10-item scale adapted from Vogel et al. (2006); scores for each item were added to create a composite score. The split sample is based on the sample mean. ^*c*^Trouble meeting monthly expenses is measured via one item: “During a typical month, how often do you worry about meeting monthlyexpenses?”.

The pride-based self-affirmation boosted how parents with high fear of judgment for help-seeking feel about themselves by 8 percentage points (*p* = 0.01) to a score of approximately 76 (out of 100). This latter rating is qualitatively similar to how parents with low fear of judgment for help-seeking rated how they feel about themselves absent the pride-based self-affirmation (i.e., those who received the neutral writing prompt). The pride-based self-affirmation also boosted how parents feel about themselves among parents earning between $30,000 and $74,999 (by 5.76 percentage points) (*p* = 0.002) and those earning $75,000 and above (by 4.21 percentage points) (*p* = 0.01). The affirmation boosted mothers’ positive feelings about themselves by 6 percentage points (*p* = 0.001) (but did not affect fathers’ feelings about themselves). Interestingly, the self-affirmation boosted mothers’ positive sense of self to the baseline levels of fathers’ positive sense of self. Additionally, it boosted positive feelings of self among parents of children of all ages and among parents who always, frequently, or sometimes have trouble meeting monthly expenses.

### Moderating Effects of the Pride-Based Self-Affirmation

Table 4 shows the effects of the self-affirmation for different levels of moderators.

**TABLE 4:** Hypothesized moderators of the impact of a pride-based affirmation on interest in parenting programs.

		Interest in learning more about a parenting program			Interest in attending a parenting program			Provided email to receive information about parenting support online resources		
	*n*	Unadjusted control group mean	β	*p*-value	Unadjusted control group mean	β	*p*-value	Unadjusted control group	β	*p*-value
**By household income^a^**										
Low	161	62.99	−0.56 (4.58)	0.90	59.47	−2.34 (4.64)	0.62	0.51	−0.06 (0.08)	0.43
Medium	400	61.90	2.71 (2.79)	0.33	56.19	2.81 (2.87)	0.33	0.48	0.00 (0.05)	0.98
High	483	58.25	10.44*** (2.38)	0.001	52.84	11.20*** (2.46)	0.00	0.35	0.10* (0.04)	0.02
**By relationship to child**										
Mother	634	61.57	4.76* (2.13)	0.03	55.51	5.16* (2.23)	0.02	0.43	0.03 (0.04)	0.17
Father	375	57.89	8.36** (2.89)	0.004	53.63	8.44** (2.88)	0.004	0.41	0.06 (0.05)	0.21
**By child age**										
Has child below age	5 498	62.92	2.94 (2.33)	0.21	57.42	2.06 (2.48)	0.41	0.45	0.01 (0.04)	0.78
Does not have child below age	5 532	58.28	7.60** (2.46)	0.002	53.00	9.00*** (2.45)	0.000	0.40	0.08 (0.04)	0.07
**By fear of judgment for help-seeking^b^**										
Low	277	65.36	3.72 (3.13)	0.24	61.16	4.23 (3.15)	0.18	0.46	0.06 (0.06)	0.31
High	235	53.20	8.97** (3.43)	0.01	47.19	10.03** (3.50)	0.01	0.35	0.13* (0.06)	0.04
**By trouble meeting monthly expenses^c^**										
Always or frequently has trouble	283	62.91	3.22 (3.30)	0.33	58.79	0.79 (3.44)	0.82	0.53	−0.01 (0.06)	0.85
Sometimes has trouble	501	61.54	6.36** (2.31)	0.01	55.99	7.21** (2.39)	0.003	0.40	0.06 (0.04)	0.17
Never has trouble	260	55.81	5.37 (3.64)	0.06	49.92	7.00 (3.65)	0.41	0.35	0.04 (0.06)	0.55
**By race/ethnicity**										
White	770	58.33	5.95** (1.97)	0.003	52.62	5.81** (2.01)	0.004	0.36	0.04 (0.04)	0.32
Black, Hispanic/Latino, Other	274	65.68	5.11 (3.17)	0.11	61.62	6.04 (3.32)	0.07	0.52	0.06 (0.06)	0.31

Authors’ calculations from data collected via participant responses to PrimePanels in April 2018. Standard errors of the coeffients are in parentheses; unadjusted control group means are based on items with scaled (0–100) responses. *p < 0.05; **p < 0.01; and ***p < 0.001. Beta (β) represents the coefficient from an OLS regression estimating the impact of receiving the pride-based affirmation on the outcome. ^Δ^ Calculated using the 25% of the sample randomized to receive the fear of judgment scale, post-randomized affirmation or control task. ^*a*^ Household income does not account for household size. Low income is below $30,000, medium income is $30,000 to $74,999, and high income is $75,000 and above. ^*b*^ Fear of judgment is measured via a 10-item scale adapted from Vogel et al. (2006); item scores summed to create a composite score. Split sample is based on the sample mean. ^*c*^ Trouble meeting monthly expenses is measured via one item: “During a typical month, how often do you worry about meeting monthly expenses?”.

#### Age of Children

The pride-based self-affirmation did not statistically significantly increase interest in learning more about (*p* = 0.21) or attending (*p* = 0.41) a parenting program among parents with at least one child under the age of 5 in the home, whereas it did significantly increase the interest in learning more about (*p* = 0.002) and attending (*p* = 0.000) a parenting program among parents with children over 5 years old by 7.6 and 9 percentage points, respectively. There was no moderating effect of age of children in the home on the relation between affirmation and parents’ submission of their email address to receive online parenting resources.

#### Race

Contrary to our expectations, the pride-based self-affirmation did not have a significant effect among parents of color on any of our outcomes of interest, however, this result is inconclusive because of large standard errors. The self-affirmation significantly increased interest among white parents in learning more, from 58 to 64 (nearly 6 percentage points; *p* = 0.003), and interest in attending, from 53 to 59 (nearly 6 percentage points; *p* = 0.004). However, the self-affirmation increased interest among white respondents to the baseline levels of interest among parents of color (i.e., those in the control group who received a neutral writing prompt).

#### Income and Trouble Meeting Monthly Expenses

The pride-based self-affirmation had impacts among parents who sometimes had trouble meeting monthly expenses, increasing their interest in learning more (by 6.4 percentage points; *p* = 0.01) and in attending parenting programs (by 7 percentage points; *p* = 0.003). A moderating effect exists for parents in the high-income category, for whom the self-affirmation increased the number of parents who submitted an email address or phone number by 10 percentage points (*p* = 0.02), compared with parents having high income in the control group.

#### Existing Fear of Judgment

Because the self-affirmation did not affect parents’ fear of judgment for help-seeking, and the results did not differ by timing of receipt of the scale (discussed later), we analyzed the scale as a moderator. Overall, parents with low fear of judgment for help-seeking reported higher rates of interest in learning about and attending parenting programs compared with parents having higher fear of judgment for help-seeking. The self-affirmation had a pronounced impact on parents with high fear of judgment for help-seeking, increasing interest in learning more about parenting programs by 9 percentage points (*p* = 0.01) and interest in attending parenting programs by 10 percentage points (*p* = 0.01). Finally, among parents with high fear of judgment for help-seeking, the self-affirmation increased the number who submitted an email address or phone number by 13 percentage points (*p* = 0.04) compared with those who had high fear of judgment and received the neutral writing prompt.

## Discussion

Fear of judgment at moments in which parents are asked about their interest in, attendance to, or subsequent practice of new parenting skills can interfere with parents’ own good intentions as well as the objectives of interventions. Social science literature shows that self-affirmations can help counter the potentially disruptive impacts of feeling threatened across a variety of domains but have not previously been tested or adapted for the domain of parenting or parent-targeted interventions. We tested the impact of a pride-based self-affirmation *via* a randomized design with a sample of parents of children age 13 or younger recruited through an online platform and found substantial increases in parents’ interest in learning more and participating in parenting programs (each with an effect size of 0.21 standard deviations) among parents who received the self-affirmation compared with those who received a neutral writing prompt.

As hypothesized, the self-affirmation appears to have influenced parents who had a high baseline fear of judgment; these parents’ ratings of interest in learning about and participating in a parenting program increased by roughly 0.34 and 0.37 of a standard deviation, respectively. These effect sizes are smaller than that of commonly used resource-intensive engagement methods, such as motivational interviewing, for which noted effect sizes range from medium to large (Coatsworth et al., 2001; Winslow et al., 2016). However, from a cost-effectiveness perspective, the self-affirmations are much less financially costly (particularly with respect to labor costs) and are scalable in real-world settings (see Gennetian, 2020 for examples). Furthermore, our findings provide evidence to support the hypothesis that pride-based self-affirmations foster a non-avoidant approach to threatening situations among those most susceptible to these threats (i.e., those with higher fear of judgment for seeking help).

### Limitations

Our randomized study had the advantage of testing self-affirmations among a large population of parents, however, the online self-report format limited our ability to measure behavioral outcomes. We found increased self-reports of interest. Our one behavioral measure—submitting an email address or phone number to receive materials—showed a pattern of favorable response in the treatment versus control group but did not reach statistical significance among the whole sample. Second, while our samples broadly map onto the national representation of parents with children, participants recruited through online platforms have some potentially unique characteristics (Stritch et al., 2017). Relatedly, because we did not specifically oversample or impose further selection criteria related to income or race/ethnicity, the extent to which we can generalize our results to low-income or non-white populations is limited. Third, our randomized study was not designed to understand how specific content or language in the self-affirmation may influence parent engagement or whether, for example, a shorter reflection exercise might have effects similar to those of a longer reflection exercise.

## Conclusion

In this study, we describe the contribution of a psychological intervention in the form of self-affirmations to parents’ self-concept and fear of judgment, and we show that self-affirmations can increase interest in parent support materials among those who might otherwise not respond or follow through. Further research is needed to better understand the extent to which self-affirmations affect behavioral measures of parent engagement ranging from show-up rates, consistent attendance, and day-to-day follow-through of new skills. Future research is also needed to better understand heterogeneity in parent engagement and determine who may be most responsive to different strategies, including different formats for delivering and experiencing self-affirmations.

## Data Availability Statement

The datasets generated for this study are available on request to the corresponding author.

## Ethics Statement

The studies involving human participants were reviewed and approved by New York University Institutional Review Board. The patients/participants provided their written informed consent to participate in this study.

## Author Contributions

All authors conceived the presented idea. ZH and MS carried out the experiment and wrote the manuscript with input from LG. LG verified the analytic methods.

## Conflict of Interest

The authors declare that the research was conducted in the absence of any commercial or financial relationships that could be construed as a potential conflict of interest.
